# Modality-specific long-term memory enhancement in *Heliconius* butterflies

**DOI:** 10.1098/rstb.2024.0119

**Published:** 2025-06-26

**Authors:** Elizabeth A. Hodge, Amaia Alcalde Anton, Louise Bestea, Greta Hernández, Jane Margareth Aguilar, Max S. Farnworth, Denise Dalbosco Dell’Aglio, W. Owen McMillan, Stephen H. Montgomery

**Affiliations:** ^1^ School of Biological Sciences, University of Bristol, Bristol BS8 1TQ, UK; ^2^ Smithsonian Tropical Research Institute, Panama City 0843-03092, Panama

**Keywords:** associative memory, Heliconiini, learning, mushroom body, olfaction, vision

## Abstract

How animals perceive, process and respond to environmental cues is tightly tuned to species-specific ecological demands and reflected by the structure of neural systems. In the Neotropical butterflies, *Heliconius*, the mushroom bodies, insect learning and memory centres are significantly expanded compared with their closest relatives. This expansion coincided with the evolution of a novel diet of pollen and the ability to learn spatial foraging routes. Previous research has shown that *Heliconius* have more accurate long-term visual memory than other Heliconiini. Here, we test whether this enhanced memory stability is specific to visual contexts by conducting a long-term olfactory memory assay in two *Heliconius* species and two outgroup species. We found no difference in long-term olfactory memory between *Heliconius* and outgroup Heliconiini, and combining data from olfactory and visual memory trials confirmed a modality-specific improvement in memory recall in *Heliconius*. Tests of how Heliconiini species prioritize olfactory and visual cues when presented in conflict show no consistent pattern, suggesting that variation in memory stability is not explained by inter-specific differences in attentiveness to sensory cues. Our data provide a rare case where memory performance has been compared across species and sensory modalities to identify evidence of a modality-specific shift.

This article is part of the Theo Murphy meeting issue ‘Selection shapes diverse animal minds’.

## 1. Introduction

How animals perceive, process and respond to the sensory world they inhabit is tightly tuned to the species-specific demands imposed by their ecology and life history. Debates concerning the evolution of cognition (broadly defined as the acquisition, processing, storage and retrieval of information) have often focused on the importance of specific or general adaptations—for example, through the relative importance of domain-general or domain-specific cognitive enhancements [1,2], or region-specific adaptations in the brain versus the importance of whole brain size [3,4] (see also [5]). The expansion of specific brain regions can provide specific cognitive advantages [6–8] and some species show adaptive shifts in specific learning and memory tasks [9,10]. However, there are few cases where test paradigms have been combined to compare memory performance across species and sensory modalities test formally whether selection has shaped the retention and recall of associative memories in a modality-specific manner.

In the insect brain, the mushroom bodies are responsible for sensory integration, learning and memory [11–14]. Intrinsic mushroom body neurons, the Kenyon cells, branch out to form the calyx, where they synapse with projection neurons carrying sensory information before extending axonal projections to form the mushroom body lobes. Across insects, differences in species’ sensory ecologies are often reflected in calyx structure and sensory input [15–18], with multisensory input occurring in many species, often within topographically separated calyx regions [16,19]. Changes in connectivity within the mushroom body are thought to be the physical basis of memory formation and they show specific effects on topographical regions of the calyx that are dependent on the sensory cue [20]. Mushroom body expansion is also observed in several insect orders, including Hymenoptera, Coleoptera, Dictyoptera and Lepidoptera [21]. Within Lepidoptera, the most substantial known expansion of the mushroom bodies occurs in the genus *Heliconius* [22,23]. These Neotropical butterflies are part of a larger tribe of butterflies, Heliconiini, that are found throughout Central and South America. Relative to the rest of the brain, mushroom body size in *Heliconius* is around four times larger than other, closely related, outgroup Heliconiini with which they share habitats and most aspects of their ecology [19,22,23]. This volumetric expansion is driven by increased Kenyon cell number and an increased proportion of calyx that receives visual input [19].

Mushroom body expansion in *Heliconius* butterflies has been linked to the evolution of pollen feeding, a novel foraging behaviour not seen in other Lepidoptera [24,25]. *Heliconius* actively collect pollen from a restricted range of plants and this provides an adult source of amino acids [26,27]. Pollen feeding in *Heliconius* is associated with cognitively demanding foraging behaviours and changes to life-history traits not seen in other genera in Heliconiini [25]. This includes the evolution of trap-line foraging, whereby long-term foraging routes are established between reliable pollen resources [28–30]. In other insects, trap-line foraging—and allocentric foraging more generally—is supported largely by memorization of visual landscape cues [31] and *Heliconius* are capable spatial learners in both field and insectary conditions [32–34]. Allied to the evolution of pollen feeding, *Heliconius* are also significantly longer-lived than other Heliconiini [24,35], with longer reproductive lifespans [36,37], creating the context for increased benefits of long-term memories (LTMs) of foraging routes [24]. Indeed, they are proficient visual learners [38] and possess enhanced long-term visual memory [19,39], which may underpin long-term recall of foraging routes that can be used for many weeks or months [28–30]. In previous comparative learning assays, all six Heliconiini species tested were able to learn colour–food associations, but only the *Heliconius* species remembered these associations after 8 days, and, in some cases, their memory persisted beyond 13 days [39]. While colour associations do not necessarily mirror the wide-field landscape cues used by *Heliconius* when foraging [39], these results suggest that the hypothesized increase in LTM of spatial cues is reflected in other visual contexts, such as colour, providing a tractable experimental system for exploring inter-specific variation in LTM across species. Our interpretation, therefore, has been that in *Heliconius*, increased stability of visual memory co-evolved with mushroom body expansion, trap-lining and the evolution of pollen feeding.

Regardless, *Heliconius* provide an interesting opportunity for comparative experiments across closely related species with specific anatomical and behavioural differences, but otherwise similar ecologies, to explore the evolution of neural and behavioural change. Here, we compare long-term olfactory memory across four species: two representatives from *Heliconius* (*Heliconius erato demophoon* and *Heliconius melpomene rosina*) and two representatives from outgroup Heliconiini (*Agraulis vanillae* and *Dryas iulia*). Although existing behavioural data strongly suggest that *Heliconius* have superior LTM in a visual context (associative colour learning) in comparison with outgroup Heliconiini [39], a generalized improvement or a concomitant shift across sensory modalities has not been tested. A specific increase in visual LTM is predicted by the demands of trap-line foraging, a context in which olfactory LTM is less relevant [38]. By mirroring Young *et al*.’s long-term visual memory experiment [39], we provide a direct comparison between long-term olfactory and long-term visual memory data to test the hypothesized modality-specific enhancement in *Heliconius*’ LTM. Using the same two *Heliconius* species (*H. erato* and *H. melpomene*) and *D. iulia* as an outgroup Heliconiini representative, we further test the capacity of Heliconiini to learn visual and olfactory cue combinations, and whether *Heliconius* prioritize visual over olfactory information when making foraging decisions in comparison with outgroup species. This allows us to explore whether variation in memory performance between sensory modalities could reflect general downregulation of attentiveness to one sensory domain.

## 2. Methods

### (a) Animal husbandry

Rearing for LTM assays was performed in January–April 2023. Butterflies of four species within Heliconiini, two *Heliconius* species (*H. erato* and *H. melpomene*) and two outgroup species (*D. iulia* and *A. vanillae*) were raised from stock populations at the Smithsonian Tropical Research Institute insectaries in Gamboa, Panama. These four species were chosen to reflect the same phylogenetic breadth and ecological differences as Young *et al*. [39], who included the same four species and one additional *Heliconius* and non-*Heliconius* species. Results from a reanalysis of Young *et al*.’s data do not change when focusing on the four species studied here, which were most amenable to rearing at the time of the experiment. Stocks of each species established from local populations were kept in 3 × 3 × 2 m mesh cages in ambient conditions and with natural lighting and were freely provided with pollen resources (*Lantana camara*, *Palicourea elata* and *Psiguria sp*.) and supplementary artificial feeders containing 20% sugar–water solution. Larval host plants were introduced several times a week to allow for egg collection. Once hatched, larvae were raised in mesh pop-up cages using the preferred host plant(s) of each species. *Passiflora biflora* was used for all four species, and *H. erato* and *D. iulia* were raised exclusively on *P. biflora. Heliconius melpomene* was raised on either *P. biflora*, *Passiflora menispermifolia* or *Passiflora riparia. Agraulis vanillae* was raised on *P. biflora* or *Passiflora tenuifila*. After eclosion, naive butterflies were moved into the experimental cages. Experimental cages contained artificial feeders and *P. elata* that had had the flowers removed. There were four cages: a pre-training cage (3 × 3 × 2 m), two training cages (2 × 3 × 2 m) and a test cage (2 × 3 × 2 m). Rearing for sensory conflict assays was performed in January–May 2024, in the same manner. These experiments include *D. iulia*, as an outgroup representative, and *H. erato* and *H. melpomene*, as representatives from *Heliconius. Agraulis vanillae* stocks were unavailable for this experiment. The cage structure, environmental conditions and origin of the butterflies (i.e. from wild-derived stocks) were consistent across experiments and with Young *et al*. [39].

### (b) Long-term olfactory memory assay

Long-term olfactory memory was assessed by invoking odour–reward associations using lemongrass (*Cymbopogon schoenanthus* steam-distilled oil; referred to here as lemongrass odour) and orange (*Citrus aurantium dulcis,* cold-pressed oil; referred to here as citrus odour) essential oil solutions (0.66% oil and 99.34% water). We used lemongrass and orange essential oils as Dell’Aglio *et al*. [40] found no evidence of a bias towards either the lemongrass or citrus odour in two species of *Heliconius*. Citrus (orange) and lemongrass scents have no known ecological relevance to any of our four species and they are, therefore, presumed to be largely neutral stimuli.

**Figure 1.**
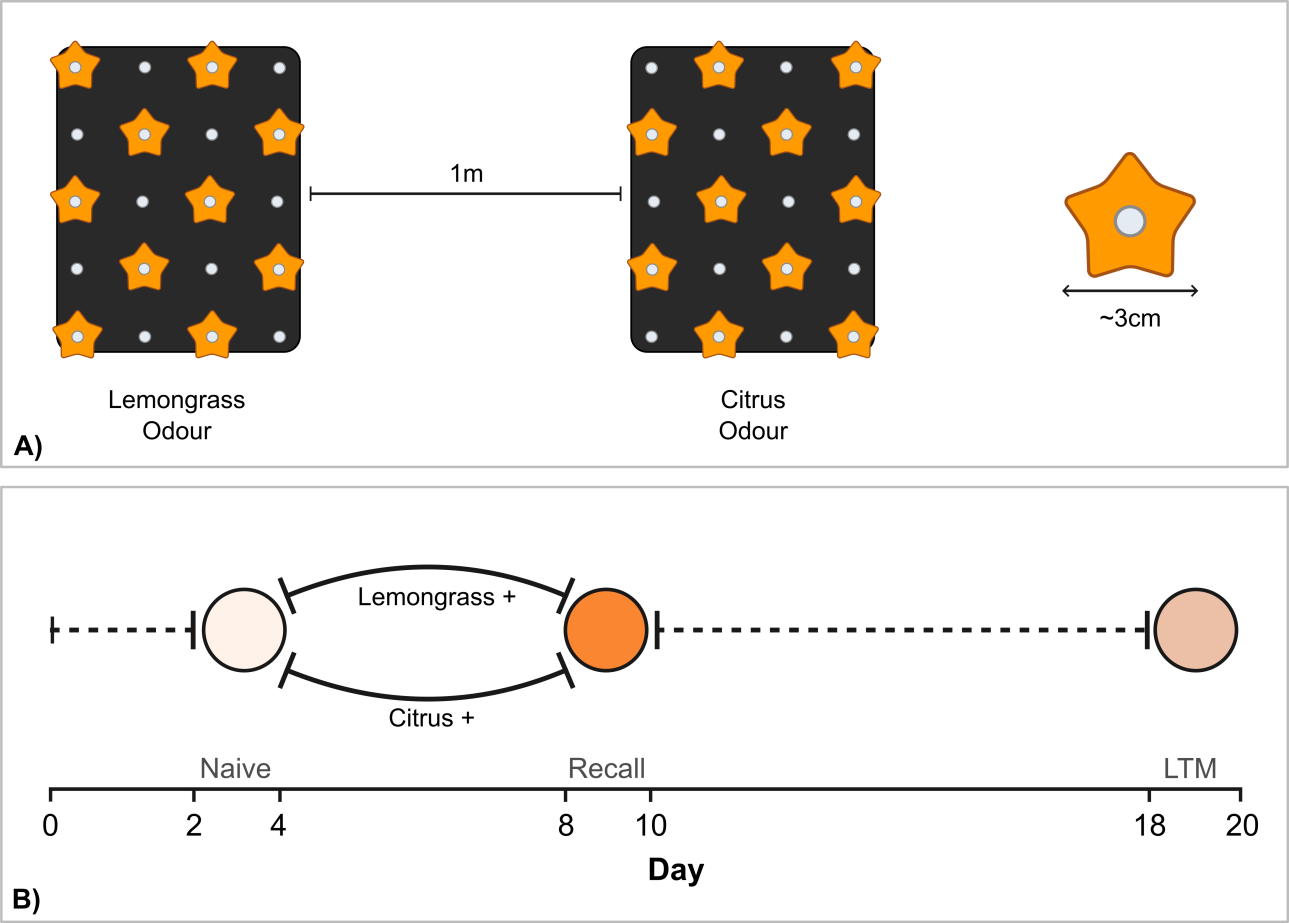
Experimental set-up (A) and protocol timeline (B) for the olfactory LTM experiment. (A) Feeder stand set-up: during the training, the artificial feeders (orange stars) are positively reinforced with sugar solution or negatively reinforced with quinine solution. Individuals were trained in groups, either inducing a positive preference for lemongrass or citrus, contra to their naive preference, i.e. the experiment was balanced, with two separate training cages where each odour was positively reinforced during training to different individuals. During the preference tests, the feeders were empty. Odour wells (white dots) between the two feeders are filled exclusively with either citrus odour or lemongrass odour solutions. (B) The bold lines indicate the training period and dashed lines indicate waiting periods when odours are not reinforced. Circles indicate the recorded preference tests.

Artificial feeders were made from orange-coloured foam star-shapes that were approximately 3 cm in diameter and had a 0.5 ml Eppendorf in the centre that could be filled with sugar–protein solution (2% critical care formula, 20% sugar and 78% water; positive stimulus) or saturated quinine solution (negative stimulus). Feeders were placed in a 4 × 6 feeder stand, with 12 feeders evenly distributed across each stand. Each artificial feeder was adjacent to at least two 0.5 ml Eppendorf tube odour wells that were filled with the essential oil solutions, providing a constant odour stimulus across the feeder stand (figure 1A). Separate feeder stands were used for each odour and were kept at least 1 m apart to minimize odour mixing.

Naive butterflies (45 *H. erato*, 51 *H*. *melpomene*, 50 *D. iulia* and 65 *A*. *vanillae*) were transferred to the pre-training cage on the day of eclosion (between 11.00 and 13.00). Each individual was sexed and assigned a unique ID, which was written on both sides of the wing in permanent marker to allow identification. The pre-training cage contained unscented artificial feeders filled with a sugar–amino acid solution. The butterflies were held in this cage for 1−2 full days after eclosion to ensure they were viable and associated the artificial feeders with a food source. After the initial pre-training period (figure 1A), the butterflies were individually moved into the test cage for a naive preference test. The test cage contained two feeder stands, one scented with the citrus odour and the other with the lemongrass odour, and empty artificial feeders. A camera (GoPro Hero 5) was mounted above each of the feeder stands for 2 h between 9.00 and 11.00 to record feeding attempts at each stand. The behavioural footage was analysed using behavioral observation researchinteractive software (BORIS) [41] to log the number of feeding attempts (defined as a butterfly probing the feeder with the proboscis) and the time at which each attempt was made, for each individual. The naive preference test was conducted across 2 days to determine which, if either, of the odours they innately preferred.

After the initial preference test period, butterflies were moved into one of two training cages depending on their behaviour in the initial preference test. In one cage, the citrus odour was positively reinforced (sugar–amino acid solution) and the lemongrass odour negatively reinforced (quinine solution), and, in the other, the lemongrass odour was positively reinforced and citrus odour negatively reinforced. Butterflies were trained on their non-preferred odour or were randomly assigned to one of the two training cages if they showed no preference in the naive test. Butterflies remained in the same training cage for four full days. On each day, the reward and non-reward solutions and odour solutions were replaced.

Following the training period, butterflies were moved back to a test cage to establish whether they had learnedt the odour–reward association (figure 1B). The recall test used the same protocol and behavioural analysis as the naive preference test. After the recall test, the butterflies were moved back to a pre-training cage, where they stayed for 8 days with no exposure to the odour stimuli. After a further 8-day period, the butterflies were moved a final time to the test cage for the LTM test, following the same protocol as the other preference tests. An 8-day period was chosen following Young *et al*.’s [39] long-term visual memory experiment protocol. Although individuals were tested in groups, behavioural data are analysed at the level of the individual. These groups included both naive individuals and individuals in the recall/LTM test trained in both directions and they were not consistent across trials (i.e. individuals were not tested in the same group in the naive, recall and LTM trials), which therefore minimized potential observer effects. We also note that *Heliconius* have previously been shown not to use social information when making foraging decisions [42].

### (c) Conflict assay

**Figure 2.**
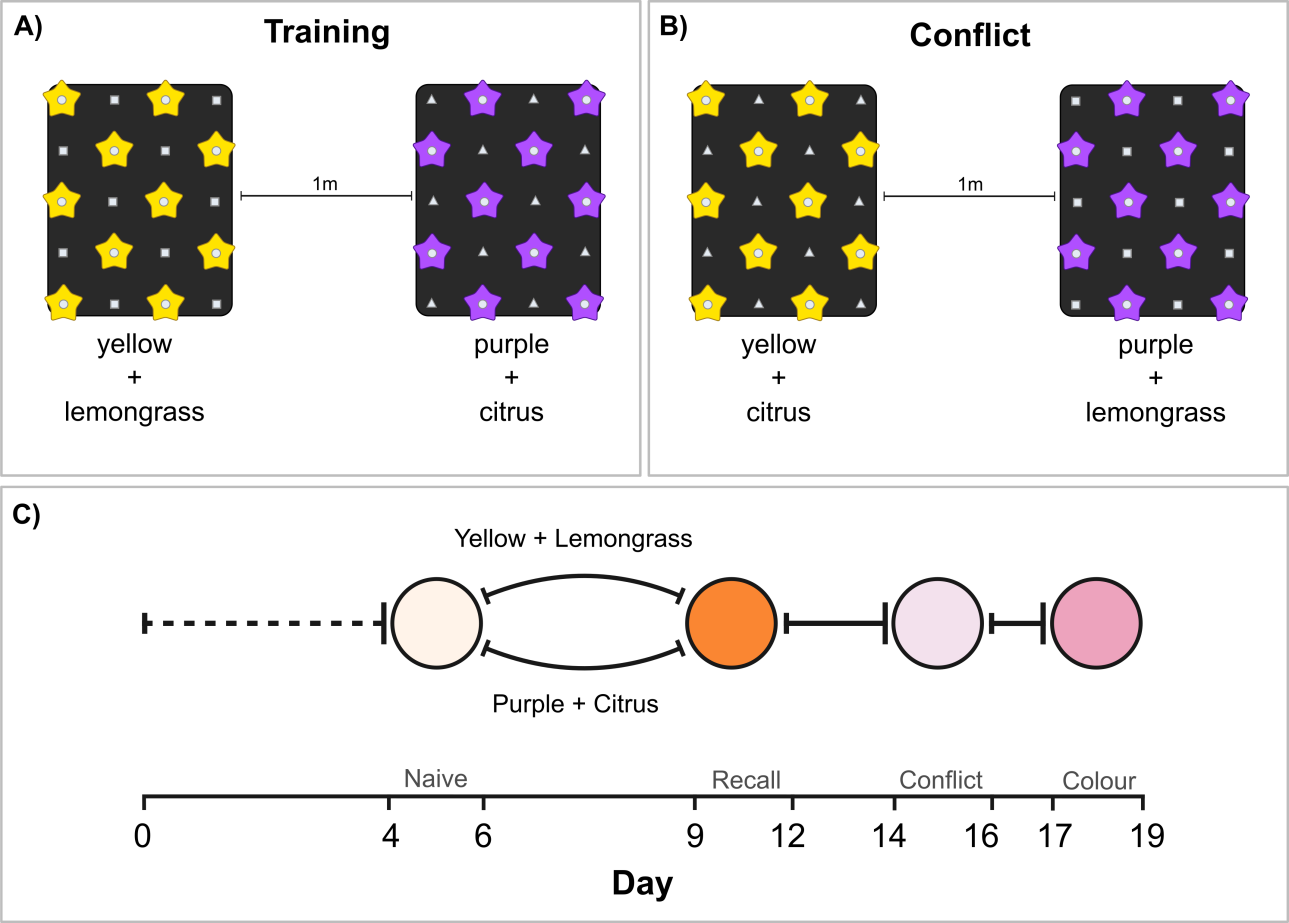
Experimental set-up for the training (A) and conflict test (B), and the timeline for the full conflict experiment (C). (A) Feeder stand set-up for the training: the artificial feeders (yellow or purple stars) were positively reinforced with sugar solution or negatively reinforced with quinine solutions, contra the naive preference test of the individual, i.e. the experiment was balanced, with two separate training cages so that each odour/colour combination could be positively reinforced during training to different individuals. The odour wells were filled with either lemongrass odour (squares) or citrus odour (triangles) solutions. (B) Feeder stand set-up for the conflict test: the artificial feeders were empty during the preference tests and the positively reinforced colour was paired with the negatively reinforced odour and *vice vers*a. (C) Solid bold lines indicate the training periods and dashed lines indicate waiting periods when odours were not reinforced. Circles indicate the recorded preference tests.

Previous protocols have explored how different *Heliconius* species weigh visual and olfactory cues following positive and negative reinforcement [40,43], based on experimental designs in prior work on hawkmoths [44]. In this experiment, we adapted these protocols to investigate the colour cues used by Young *et al*. [39] in combination with the long-term olfactory memory test odour cues above (figure 2). The experiment used naive butterflies. After eclosion, butterflies were sexed and ID-ed before being placed in a waiting cage. In the waiting cage, each butterfly was trained to feed from white artificial feeders so that they recognized these as a food source. Butterflies remained in the waiting cage for 1–3 days post-eclosion before being transferred to the naive preference test (figure 2C). As sensory cues, we used artificial flower feeders made of five-pointed foam stars in two different colours, yellow and purple, with a 0.5 ml Eppendorf tube in the centre, containing either the positive (1% pollen, 20% sugar and 79% water solution) or negative (saturated quinine solution) reinforcement (figure 2A). Similarly to the long-term olfactory memory assay, the feeders were evenly distributed across a 6 × 4 feeder stand, which could be introduced and removed from the experimental cages as needed. As an olfactory cue, the feeder stands had evenly distributed odour wells, which contained one of two essential oil solutions that were also used in the previous experiment: lemongrass (*C. schoenanthus,* steam-distilled oil) and orange (*C. aurantium dulcis*, cold-pressed oil), following Dell’Aglio *et al*. [40]. Each essential oil solution was prepared with 0.66% oil and 99.34% water. This coupled the visual cue of the feeder with a particular odour, such that one combination was positively reinforced and one was negatively reinforced. As in the LTM experiment, the tests were recorded using two cameras (GoPro Hero 5) and the videos were annotated using BORIS software [41].

After 4−5 days in a waiting cage, naive butterflies (53 *H. erato*, 20 *H*. *melpomene* and 37 *D. iulia*) were exposed to two different colour–odour combinations: yellow+lemongrass and purple+citrus. The feeders were left empty and butterfly behaviour was recorded for 2 h to check for individual, naive preferences. After this test, the butterflies were trained for three full days against their initial preference, such that in the dataset, the experiment was fully balanced with colour/odour combinations presented with both valences (i.e. CS+ and CS−; figure 2C). The butterflies were then transferred to a training cage where their initial preference was negatively reinforced, while the non-preferred combination was positively reinforced. If a butterfly did not exhibit a clear preference, one of the training combinations was randomly assigned. After the training period, the butterflies were returned to the test cage and a recall test was conducted following the same protocol as the naive preference test. This recall test aimed to determine whether the butterflies had learned the associations during the training period. After the recall test, butterflies were moved back to their training cages for an additional 1–2 days to further reinforce the colour–odour associations. On the 14th/15th day, a conflict test was performed in which the positively reinforced colour was paired with the negatively reinforced odour, and *vice versa*, so the new combinations were as follows: yellow+citrus and purple+lemongrass (figure 2B). Following this, butterflies underwent an additional day of the same training that they had in the beginning. Finally, a colour preference test was conducted to assess whether the butterflies had learned the odour and colour cues independently (figure 2C). In this final test, only purple and yellow feeders were presented, with no associated odours.

### (d) Statistical analysis

In the LTM assay, as we were interested in retention of the trained association, individuals that did not successfully learn during the training period (accuracy <50%) were removed from the dataset. This resulted in the removal of 37 individuals: 10 of 65 *A*. *vanillae*, 13 of 50 *D. iulia,* 8 of 45 *H*. *erato* and 6 of 51 *H*. *melpomene*. We repeated the analyses with higher thresholds (60 or 70% accuracy) and similar results were obtained (electronic supplementary material, tables S6 and S7). Analyses of feeding preference were then carried out using generalized linear mixed models (GLMMs) with a binomial distribution in R v.4.3.0 [45] using the *glmer* function in the *lme4* package v.1.1-33 [46]. When looking at inter-specific differences, *species* and *trial* (naive test, recall test and LTM test) were treated as fixed effects. We included the interaction term between *trial* and *species*, despite its lack of significance, because it was of interest to us. When looking at intra-specific differences, only *trial* was used. To determine whether feeding attempts for the two odours were random in the naive preference test and the LTM test, deviation from 50% was assessed using a null GLMM (intercept-only model including no fixed effects). The long-term olfactory memory protocol followed Young *et al*.’s 2024 visual LTM experiment [39] as closely as possible, including in experimental design and in animal husbandry (see above). Young *et al*.’s [39] data included using four of the same populations/species to allow comparisons between visual and olfactory LTM performance. The data for both experiments were combined and differences in the species’ ability to learn and retain olfactory versus visual associations were analysed using GLMMs. Inter-specific differences were analysed using species, trial and experiment (visual or olfactory) as fixed effects and ID and observation-level random effects to account for overdispersion. Additionally, differences in performance between the *Heliconius* genus and outgroup Heliconiini group were analysed, also with trial and experiment as fixed effects.

In the conflict experiment, we aimed to assess the preference for odour or visual cues after the butterflies had learned a colour–odour association. Therefore, we excluded individuals that did not successfully learn this association, resulting in the removal of four individuals: 1 of 20 *H*. *melpomene* and 3 of 37 *D. iulia*. During the video analysis, we observed that a few butterflies landed on the odour Eppendorf odour wells rather than the artificial flowers, seemingly attracted to the source of the odours. We therefore created the following two datasets: one accounting for these odour-well landings (included in §3 and figure 5) and one that focuses solely on feeding attempts at the artificial flower (included in electronic supplementary material, figure S3, tables S12 and S13). To analyse the conflict data, we used GLMMs with a binomial distribution to analyse cue preference using the *glmer* function from the *lme4* package v.1.1-33 [46]. To build the model, we began with the most complex model and progressively simplified it based on the results of ANOVAs between models, removing the least significant fixed factor each time. We use the proportion of attempts on the trained colour (taken as performance) as the response variable. Initially, the model included fixed factors for *trial* and *species* and ID as random factor. We also included the interaction term between *trial* and *species*, despite its lack of significance, as it was of interest to us. We built additional models to assess whether the proportion of feeding attempts on the trained colour was significantly different from 50%. As above, we created a null GLMM as an intercept-only model including no fixed effect. Finally, we built another model to check for differences in the number of feeding attempts per individual, using a GLMM with a Poisson distribution in this case.

For both experiments, model diagnostics were performed using the *DHARMa* package v.0.4.6, and ID and, where necessary, observation-level random effects were included as random effects to account for overdispersion, which occurs when the variance of the response variable is greater than expected. Including observation-level random effects improved the model fit. The package *emmeans* v.1.8.6 [47] was used for *post hoc* pairwise comparisons between species and trial, using the estimated marginal means with Tukey correction for multiple comparisons.

## 3. Results and discussion

### (a) Proficient long-term memory of olfactory associations across Heliconiini

**Figure 3.**
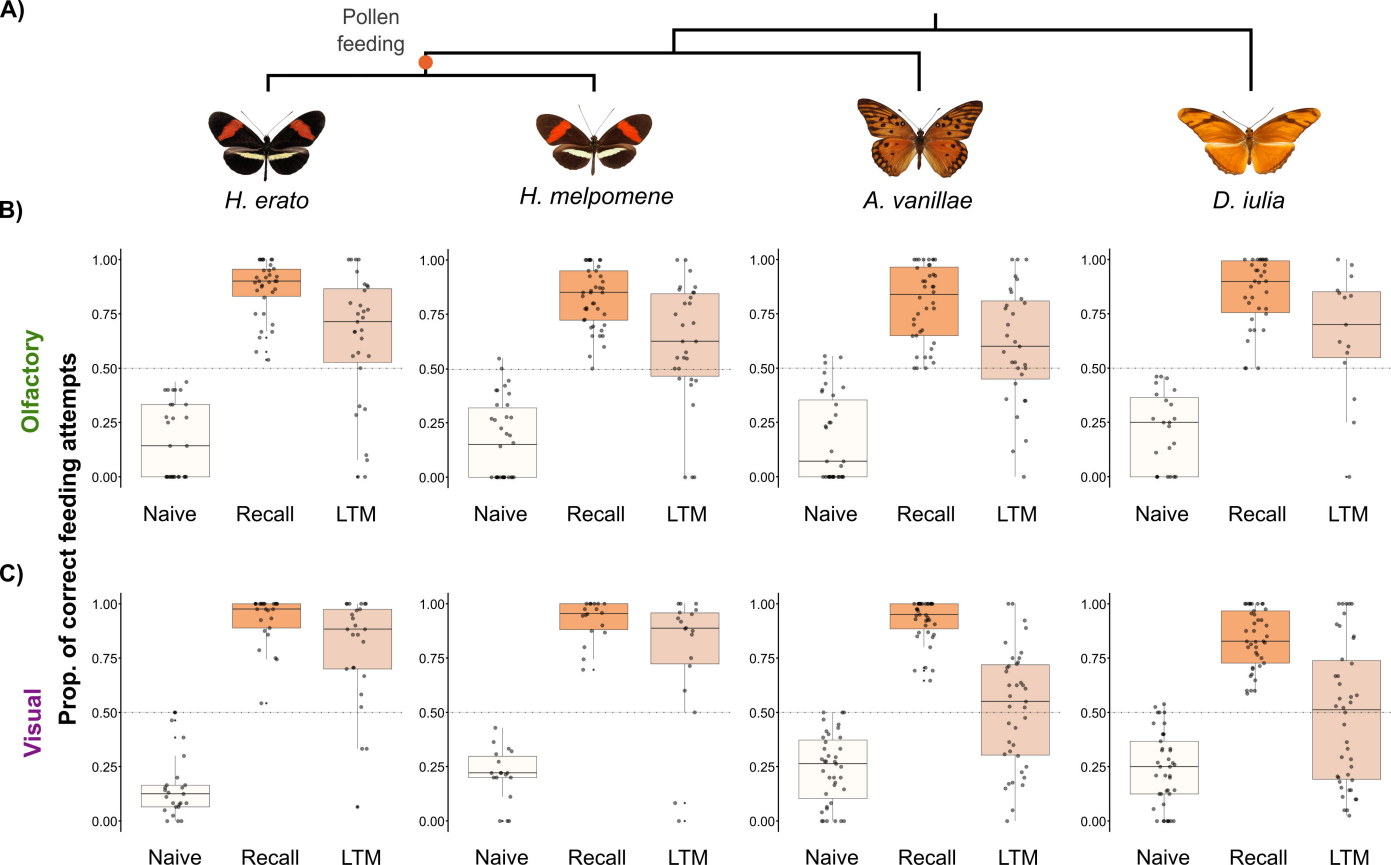
(A) Phylogeny of species used in the behavioural assays (pollen feeders: *H. erato, H. melpomene*; non-pollen feeders: *A. vanillae* and *D. iulia*). (B/C) Performance across long-term olfactory (B) and visual (C) memory experiments, colour-coded by trial: white = naive preference, dark orange = recall test, light orange = LTM test after 8 days of no reinforcement of the trained cues. Data for the visual trials are taken from Young *et al*. [39].

*Heliconius* possess expanded mushroom bodies approximately 4 times larger, relative to total brain size, than their outgroup genera *Dryas* and *Agraulis* [19]. Here, we compared olfactory memory across two *Heliconius* species (*H. erato* and *H. melpomene*) and two outgroup species (*D. iulia* and *A. vanillae*). There was no significant variation between the species in their naive odour preferences (electronic supplementary material, figure S1; *χ*^2^ = 4.016, *df* = 3, *p* = 0.258). *H. melpomene, D. iulia* and *A. vanillae* all showed modestly biased preferences towards the citrus odour (electronic supplementary material, tables S1, S2 and figure S1), but the spread of data covered the full range of feeding preferences in all species. All four species were able to learn the odour associations with high fidelity (electronic supplementary material, figure S2)**,** showing significant shifts in preference between the naive test and the recall test (*χ*^2^ = 400.817, *df* = 1, *p* < 0.001; figure 3A; electronic supplementary material, table S3). There were no differences between the species’ abilities to learn the odour associations (*χ*^2^ = 3.024, *df* = 3, *p* = 0.388), with all species learning the association with similar accuracy. Compared with their performance in the recall test, all species showed a decline in accuracy in the LTM test after the 8-day period without exposure to the odour cues (*χ*^2^ = 57.398, *df* = 1, *p* < 0.001; figure 3A; electronic supplementary material, table S3). This suggests some memory loss; however, all four species showed accuracy greater than chance, suggesting functional long-term olfactory memory lasting over a week (electronic supplementary material, table S4; figure 3A). There were no inter-specific differences in LTM performance (*χ*^2^ = 2.445, *df* = 3, *p* = 0.485) and belonging to the *Heliconius* genus did not equate to greater ability to recall the olfactory association (*χ*^2^ = 0.280, *df* = 1, *p* = 0.597).

Visual cues are thought to be the primary sensory modality exploited during the derived foraging behaviour of *Heliconius*. Nevertheless, olfactory cues are important for all Heliconiini during intra-specific communication, mate choice and detection of close-range floral and host plant cues [48–51]. Our results build on existing evidence of olfactory learning in Heliconiini species: learning of artificial odour cues shown in *H. erato* and *Heliconius himera* [40] and recognition of vegetative and floral odours shown in *H. melpomene* [52]. The mushroom bodies’ role in the formation of olfactory memories and their importance for olfactory processing in insect species are widely recognized [11,20,53,54]. The expansion of *Heliconius* mushroom bodies is primarily driven by an increase in the volume of calyx receiving input from visual projection neurons, with a much lesser expansion of the olfactory calyx. Indeed, the raw volumes of olfactory calyx overlap between *Heliconius* and outgroup genera, unlike the visual calyx volumes [19]. This indicates a degree of visual specialization in the *Heliconius* mushroom bodies, supported by previous evidence showing increased stability of visual memories in *Heliconius* compared with outgroup Heliconiini [39]. The absence of enhanced olfactory memory may, therefore, suggest a modality-specific memory enhancement that mirrors neuroanatomical variation across the tribe.

### (b) Long-term memory recall varies across sensory modalities between pollen-feeding and non-pollen-feeding Heliconiini

**Figure 4.**
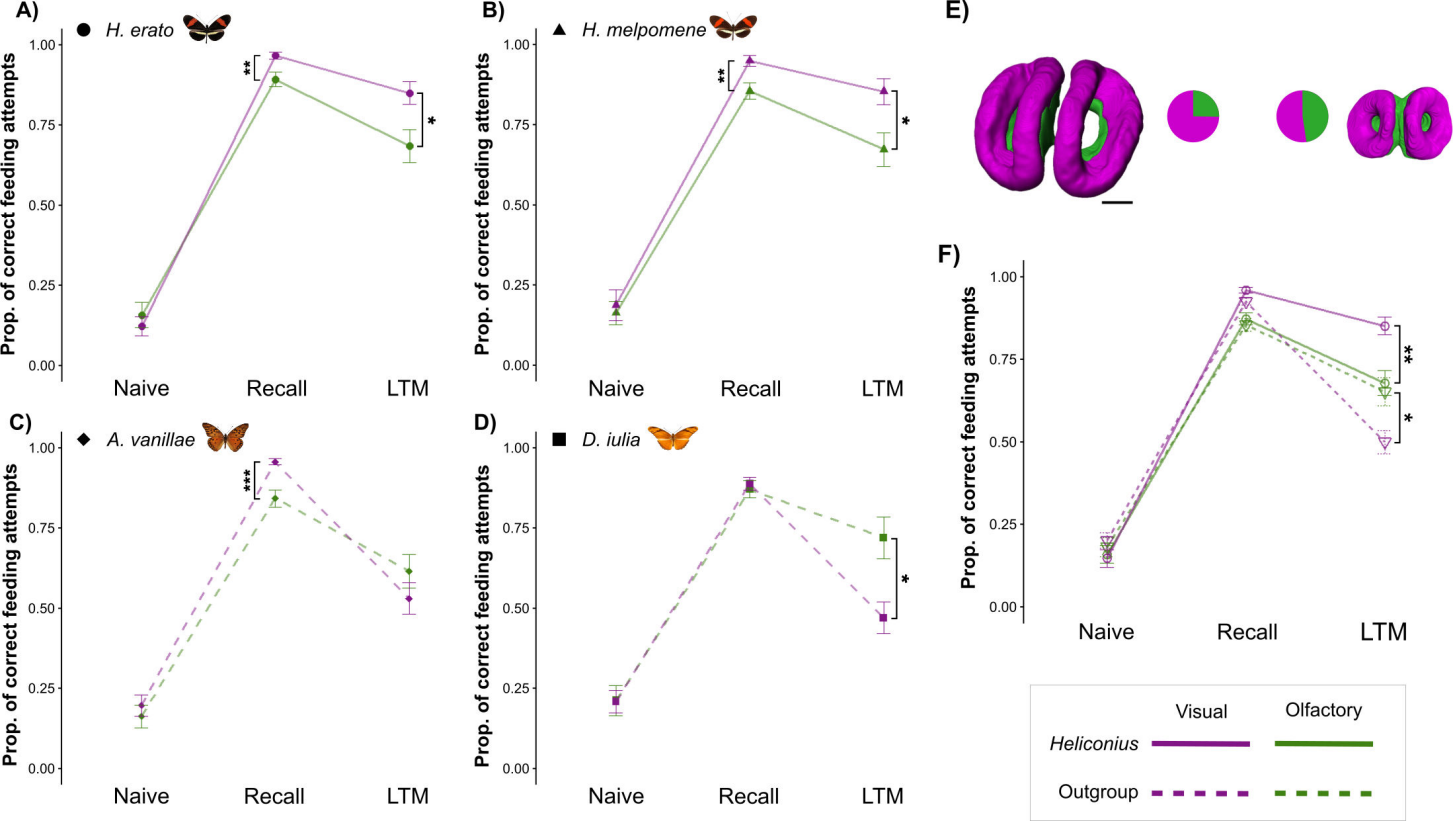
(A–D) Differences in performance across the three trials (naive, recall and LTM) in the visual (magenta) and olfactory (green) experiments for each species. Values, including standard errors, extracted from coefficients of a GLMM including a significant *Species × Trial × Experiment* interaction. Asterisks indicate significant pairwise differences in performance between the visual and olfactory experiments. *Heliconius* species are shown with solid lines and outgroup Heliconiini species with dashed lines. (E) 3D models of the mushroom body calyx showing the visual processing areas (magenta) and olfactory processing areas (green) in a representative *Heliconius* and outgroup, with their relative proportions shown in pie charts. Scale bar = 100 μm. (F) Pooled *Heliconius* species performance (circles with solid line) compared to pooled outgroup Heliconiini species (triangles with dashed line) between the visual (magenta) and olfactory (green) experiments, showing the shift in memory fidelity in *Heliconius*.

To formally test the hypothesized modality-specific shift in memory, we combined data (electronic supplementary material, table S5) on performance in the olfactory and visual experiments (from [39]). We found no differences in behaviour between the *Heliconius* and outgroup butterflies in the naive tests for either experiment (electronic supplementary material, table S6). In contrast, we found sensory modality-specific effects on learning and memory. All four species demonstrated comparable or more accurate visual learning than olfactory learning (*χ*^2^ = 28.331, *df* = 1, *p* < 0.001; electronic supplementary material, tables S6 and S7). *Heliconius*’ long-term visual memory was also superior to their olfactory LTM (electronic supplementary material, table S6; figure 4) and to the long-term visual memory of the outgroup genera (as reported in [39]; electronic supplementary material, table S6). In contrast, the outgroup genera had superior olfactory memory to visual memory (electronic supplementary material, table S6) and no group differences were found in olfactory memory (*χ*^2^ = 2.579, *df* = 1, *p* = 0.612; figure 3B). These group-level effects are reflected in patterns of intra-specific performance (electronic supplementary material, table S7; figures 3B,C and 4). Both *Heliconius* species were more accurate in the visual recall test than in the olfactory recall test and had more accurate long-term visual memory than long-term olfactory memory (electronic supplementary material, tables S6 and S7; figure 4). In contrast, there were no differences in accuracy in learning olfactory versus visual cues for *D. iulia* (electronic supplementary material, table S7)*,* which showed higher accuracy in the olfactory LTM test (figure 4). In the recall test, *A. vanillae* were better at learning visual associations than olfactory associations but performed equally well in both LTM tests (electronic supplementary material, table S7). While the intensive nature of performing these experiments across multiple, free-flying species restricts the range of stimuli used, we focused on ecologically neutral odours and equally non-preferred colours. Given that learning performance in the recall test was largely consistent across species and the salience of each association with the reward was equal, we argue that the learned associations are typical of general performance.

### (c) No consistent shifts in prioritization of visual and olfactory cues during foraging decisions by Heliconiini

**Figure 5.**
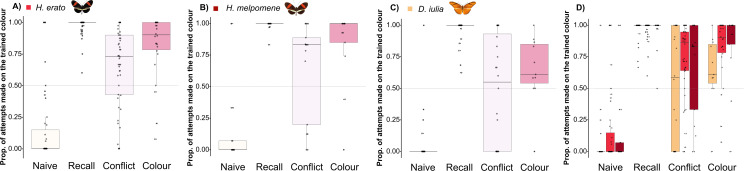
Proportion of feeding attempts made on the trained colour by *H. erato* (A), *H. melpomene* (B) and *D. iulia* (C) across the different trials. The graphs are colour-coded by test: white for naive preference, dark orange for the recall test, light pink for the conflict test and pink for the colour-only test. In (D) colour-coding is by species, orange for *D. iulia*, light red for *H. erato* and red for *H. melpomene*, to compare the performance of the three species across trials.

We next tested how Heliconiini butterflies prioritize visual or olfactory cues when positively reinforced visual and olfactory cues were presented in conflict. This acts as a test of whether the apparent shift in visual memory stability was specific to learned preferences or reflected changes in sensory attention between *Heliconius* and other Heliconiini. All three species (*H. erato, H. melpomene* and *D. iulia*) successfully learned a colour–odour association when trained against their initial preference (see recall test in figure 5; electronic supplementary material, tables S8 and S9). The cues were subsequently presented in conflict, with the positively reinforced colour now paired with the negatively reinforced odour, and *vice versa*. With these conflicting cues, there were significant differences between the proportion of feeding attempts made towards the positively reinforced colour between the recall test and the conflict test (electronic supplementary material, table S8). This suggests that both visual and olfactory cues are important when making foraging decisions in Heliconiini. We also observed an increase in the variability of response, which likely reflects a greater degree of ‘uncertainty’ during decision-making. In the conflict test, the proportion of feeding attempts on the positively reinforced colour was significantly above 50% in *H. erato*, suggesting a preference for visual cues over olfactory ones (electronic supplementary material, table S10). A similar trend was observed in *H. melpomene* butterflies, although this was not significant (electronic supplementary material, table S10; figure 5). In the case of *D. iulia*, there appeared to be no preference for either cue (electronic supplementary material, table S10). However, overall, we found no significant differences between species during the conflict test (electronic supplementary material, table S9). Significant differences were observed between the conflict test and the colour test for *Heliconius* butterflies, but not for *D. iulia* (*H. erato*: estimate = −1.334, *z*. ratio = −2.435, *p* = 0.040; *H. melpomene*: estimate = −2.157, *z*. ratio = −2.440, *p* = 0.039; and *Dryas*: estimate = −0.766, *z*. ratio = −0.922, *p* = 0.626; electronic supplementary material, table S8). Finally, *Heliconius* also tended to perform better during the final colour test, where the odour cues were absent, but this difference was not significant (electronic supplementary material, table S9; figure 5). We also note that *D. iulia* individuals made significantly fewer feeding attempts per individual in the later trials, particularly during the conflict test (*D. iulia* versus *H. erato*: estimate = −1.28, *z*. ratio = −5.627, *p* < 0.001 and *D. iulia* versus *H. melpomene*: estimate = −1.211, *z*. ratio = −4.332, *p* < 0.001; electronic supplementary material, table S11), which may indicate greater uncertainty in decision-making. Overall, these results suggest that both visual and olfactory cues are extracted by Heliconiini butterflies and used with similar importance across genera. While there is some suggestion that *Heliconius* may rely more on visual cues when learning colour–odour combinations, this was not sufficient to drive differences in performance when the cues were presented in conflict.

### (d) Visual specialization of memory performance and circuitry in *Heliconius*

Our results (summarized in electronic supplementary material, figure S4) support the hypothesis that increased investment in visual processing in the mushroom body calyx is linked to specific, derived behaviours of *Heliconius*. It is likely that, when trap-lining, *Heliconius* use a combination of sensory stimuli but rely on visual cues for navigation and continued daily use of foraging routes [32,39]. As these routes can be utilized for many weeks or months [25,30,55], it is likely that *Heliconius* may have experienced increased selection for greater retention of visual LTMs. While colour learning is a distinct context from the learning of landscape cues involved in spatial foraging [39], it provides more experimental tractability and has previously been shown to detect inter-specific variation between pollen-feeding *Heliconius* and their non-pollen-feeding Heliconiini relatives [39]. Data presented here suggest that this is a modality-specific memory enhancement, with increased long-term stability of visual memories, but not olfactory memories. Our conflict experiments further suggest that this occurs in the absence of a consistent shift in how visual and olfactory associations are prioritized during memory formation, implying that the greater retention of visual memories in *Heliconius* is unlikely to be owing to any shift in sensory reception.

Our conclusions align with both the inferred conservation of sensory pathways across pollen-feeding and non-pollen-feeding Heliconiini (Hodge *et al.*, in prep [56]). Conservation in sensory pathways implies that an isolated pattern of change in the mushroom bodies is associated with cognitive evolution in *Heliconius* butterflies and with the specific expansion of visual processing regions of the mushroom body calyx, which dominate the volumetric increase observed in *Heliconius* [19]. *Heliconius* have a sixfold to eightfold increase in Kenyon cell number and, while the proportion of cells receiving visual versus olfactory input has not been clearly delineated, it is likely that these additional cells predominantly receive visual information. Evidence also suggests that a change in the degree of post-eclosion synaptic plasticity accompanies this Kenyon cell increase. *Heliconius* have a greater degree of, and environmental sensitivity to, synaptic pruning compared with *D. iulia* [39]. In Hymenoptera, synaptic plasticity in the calyx is linked to LTM formation [20] and it occurs in a sensory modality-specific manner, with olfactory experience predominantly affecting the Hymenopteran calyx lip, while visual experience affects the collar [15,20]. Whether the improved visual LTM in *Heliconius* is directly linked to increased Kenyon cell number or plasticity remains to be determined. However, the absence of detectable changes in upstream sensory pathways [19] strongly implies that these behavioural changes are based on altered mushroom body function.

## 4. Summary

To our knowledge, we have provided a rare example of formal experimental evidence of an evolved, memory enhancement that is specific to certain sensory modalities in any insect. This specificity reflects the capacity for selection to drive changes in specific dimensions of learning and memory, rather than being reliant on modality-general mechanisms for increasing LTM performance. This could be beneficial in some contexts, perhaps owing to the high cost of LTM [57]. Given that long-range navigation of learned spatial routes is supported by visual cues in insects [58,59] and the likely greater stability of landscape cues compared with olfactory plumes in tropical forests, we suggest that the enhanced LTM of colour cues reflects variation in learning across the visual domain and is adaptive for *Heliconius* because it improves foraging efficiency, while similar changes in olfactory memory would be less beneficial. Regardless of the ecological context for these changes in memory, combined with neuroanatomical evidence for visual specialization of the mushroom body (electronic supplementary material, figure S4), our results provide support for claims that selection shapes specific aspects of cognitive performance to meet a species’ learning and memory needs [60].

## Data Availability

All data and code are provided in the zip file, and are available at [61]. Supplementary material is available online [62].
