# Correction: ‘Modality-specific long-term memory enhancement in *Heliconius* butterflies’ (2025), by Hodge *et al*.

**DOI:** 10.1098/rstb.2025.0262

**Published:** 2025-08-21

**Authors:** Stephen H. Montgomery

**Affiliations:** ^1^ School of Biological Sciences, University of Bristol, Bristol BS8 1UG, UK

**Keywords:** associative memory, Heliconiini, learning, mushroom body, olfaction, vision

*Phil. Trans. R. Soc. B* **380**, 20240119. (Published online 26 June 2025). (https://doi.org/10.1098/rstb.2024.0119)

An author’s name was incorrectly spelt in the original publication. An author was listed as Jane Margereth Aguilar in the original version, which has been corrected to Jane Margareth Aguilar.

